# Pitfalls and technical errors in the first approach to neonates with anorectal malformations in a non-specialist context: can we do any better? A review from three Eastern African Centres

**DOI:** 10.4314/ahs.v21i3.45

**Published:** 2021-09

**Authors:** Alessandro Calisti, Faisal Abdelgalil Nugud, Kibreab Belay, Agnes Mlawa, Pierluigi Lelli Chiesa

**Affiliations:** 1 Pediatric Surgery – San Camillo-Forlanini Hospital – Rome – Italy; 2 Gezira National Centre for Pediatric Surgery – Wad Medani – Sudan; 3 Orotta National Referral Hospital – Asmara – Eritrea; 4 Consolata Hospital Ikonda – Makete – Tanzania; 5 Pediatric Surgery Unit, Hospital “Santo Spirito” of Pescara and University “G. d'Annunzio” of Chieti Pescara, Pescara, Italy

**Keywords:** Ano rectal malformations, neonate, low resources context

## Abstract

**Introduction:**

In sub-Saharan Africa, Anorectal malformations (ARM) are the most frequent cause of neonatal obstruction. Referral to a Pediatric Surgeon is frequently delayed. The first treatment is often delivered at not specialist level and mismanagement may result.

**Aim:**

To study ARM patients referred beyond neonatal period and managed at a non-specialist level.

**Materials and Methods:**

One hundred and thirty patients were included (M/F ratio 63/67) among 144 admitted to three Eastern African Hospitals with Pediatric Surgical facilities. Demographics, type of anomaly, delay on referral, previous management, most commonly observed errors are reported.

**Results:**

The Mean age at referral was 23 months (range five weeks – 23 years). Colostomy was the most frequent surgery (92 cases). Stomas often did not follow the recommended criteria. Ten per cent were not on the sigmoid, and 35% were not divided. “Loop” or “double-barrel” colostomies did not exclude the distal loop. Inverted (10,5%), prolapsed stomas (7,5%), short distal loop (16%) were observed. Twenty-four cases (26%) needed redo. Primary perineal exploration in eight patients resulted in incontinence.

**Conclusions:**

Investments on training practitioners, acting at District/Rural level, and closer links with tertiary centres are recommended to avoid ARM mismanagement and delayed referral to a Specialist.

## Introduction

Reduced access to care for neonatal surgical conditions is a common problem in sub Saharan Africa, where most of the deliveries happen outside hospitals^1^. Lack of transportation associated with diffuse poverty hampers referrals from extra-urban and rural Areas to the few established Specialist health facilitieshe number of paediatric surgeons is far below the needs^2^. Neonates first referred to a primary or secondary level health facilities are generally managed by adult surgeons with few experiences in paediatric diseases.

Anorectal Malformations (ARM) are the most frequent (57%–67%) neonatal intestinal obstruction in Africa and the commonest (up to 64%) reason for neonatal surgical referral^3^–^4^–^5^–^6^. Male neonates survive after an emergency colostomy for bowel obstruction which is done, in many cases, at a district or rural hospital by a non-specialist practitioner. In a small number of cases, a blind perineal surgical exploration is sometimes attempted trying to create an anal opening at the site of the visible anal dimple. The first surgical approach in a non-specialist contest frequently did not follow the recommended criteria for ARM^8^ and may severely affect the definitive repair. Female cases can also stay undiagnosed passing stools through recto vestibular or vaginal fistula until adult life.

Patients with ARM are not infrequently (from 19-to 85% according to Lawal)^7^ referred to a tertiary Centre far beyond the neonatal period for definitive repair by a Posterior Sagittal Ano Recto Plasty (PSARP). The present study aims to warn about delayed referral and possible mismanagement of neonates with ARM at a non-specialist contest and to suggest possible solutions.

## Material and Methods

The study was done in three Eastern African Health Centres with Pediatric Surgical facilities within the framework of a research partnership with Italian Teaching Hospitals. At the Orotta National Referral Hospital (ONRH) of Asmara, Eritrea, it lasted 43 weeks and concluded in 2011, at the Gezira National Centre for Pediatric Surgery (GNCPS) of Wad Medani, Sudan it lasted 30 weeks and ended in 2020. At the Consolata Hospital Ikonda (CHI), a charitable Health institution in the district of Makete, Tanzania, it lasted 27 weeks and concluded in 2019.

The three Institutions are representative of different paediatric surgical contexts in East Africa:

a) In Eritrea, Orotta Hospital is the only national referral place, with main medical specialities represented, serving a relatively small country (117,600 km^2^) where about six million of people live. Major neonatal and paediatric cases, must be all referred to Orotta for treatment from the district and rural hospitals. Outcome is frequently conditioned by delay in diagnosis and referral or inappropriate early management. A postgraduate training in surgery, mainly orientated to adult conditions was started in Eritrea in 2007 but exposition of trainees to paediatric cases is occasional and conditioned by poor intensive care facilities. The level of care for surgical neonates and children at a peripheral level remains low. Paediatric surgical coverage is currently provided in Asmara, by several international outreach initiatives that are still building “on the job” a limited local specialist workforce and providing a basic paediatric training to a few general surgeons.

b) At the GNCPS of Wad Medani there Is a different situation. Pediatric surgery in Sudan was first recognized as an independent medical specialty in 1972, and currently, there are specialist paediatric surgeons. Eleven are based in Khartoum hospitals, and four are active at the GNCPS, in the Gezira state, where only four out of a total of sixty million of inhabitants of Sudanese live. This limited workforce cannot cover the needs of a country of 188,000 square kilometres. The level of training is good but the number of new entering specialists is reduced by a constant “brain drain” toward better salaries and professional opportunities abroad. A large number of children finds obstacles accessing the few specialist centres due to lack of transportations from far distant areas and poverty. In most of the cases life-threatening or complex conditions are primarily managed at a district/rural level by a surgeon who, as a trainee, had a very low exposition to paediatric cases.

c) Consolata Hospital's connections to the nearest specialist tertiary centre are eight hundred kilometres of not all-weather roads served by bad quality transports. This condition is very common in Tanzania. The country has still a small Pediatric Surgical workforce, estimated in only 12 surgeons in 2019, all working in two tertiary Centres, for a population of about 22 million under 15 years old. An academic paediatric surgical curriculum and training started since 2002 supported by international academic partnerships in order to fill the gap through various modalities (short courses, informal training for medical students, formal master-level training). Most paediatric cases risk so far to fall under the coverage of general surgeons not exposed to paediatric surgery as a specialist field during their undergraduate training and internship. The quality of care may be severely affected. Consolata Hospital's paediatric surgical service started in the last four years under the coaching of an outreach team. Cases are referred from other hospital of Njombe Region where 702,097 people live scattered on 41,311 squarekilometers. All three hospitals receive, for a different reason, a certain number of ARM patients beyond neonatal age after a first treatment undertaken in a peripheral Healthcare facility. We selected for the purposes of the present study only these cases. Data about demograhics, age at referral, type of ARM, associated malformations, kind of treatment received according to gender, type of procedure, related complications or sequelae have been analysed and reported.

## Results

**Demographics:** One hundred and thirty of 144 ARM patients (90%) observed in the three Institutions, met the criteria of the study (79/94 GNCPS, 39/40 ONRH, 11/11 CHI). Male to female ratio was 63/67. Mean age at referral was 23 months (median 10, range five weeks – 23 years, SD 37,5) without any significant difference between males and females.

B) Type of ARM: Table I reports different types of ARM observed, according to Krickenbeck Classification.

**Table I:** Krickenbeck Classification of 130 ARM cases

	M	F
*Perineal (cutaneous) fistula*	*6*	*1*
*Rectobulbar fistula*	*48*	
*Rectoprostatic fistula*	*3*	
*Rectovesical fistula*	*1*	
*Recto Vestibular fistula*		*55*
*H Type Rectovaginal fistula*		*3*
*Cloaca*		*7*
*Rectal atresia*	*4*	*1*
*Colon Pouch*	*1*	

C) Associated anomalies: Seven cases (5.3%) presented an associated congenital abnormality or a chromosomal defect.

D) Previous treatment in Males: Fifty-seven males had a functioning colostomy made at birth (90.4%). In four of them, a surgical exploration of perineum had also been done, and an incontinent or stenotic faecal fistula resulted. Mean delay between a colostomy and the admission for a final ARM repair was twenty-four months (range 5 weeks-17 years, median 12 months, SD 35,6). Six patients came without a colostomy. Three of them were passing stools through a misplaced perineal surgical opening done as first treatment at birth. Urine leakage from a rectourethral fistula was noted in one of them. The other three male patients had been passing stools since birth through a rectoperineal thigh fistula and presented severe constipation. One of them was 12 months old on referral.

E) Previous treatment in Females: Fifty-two per cent of females with ARM had a colostomy done in the first days or weeks of life. In one of them, a cut back of a recto vestibular fistula had resulted in a patent, incontinent anal opening. The mean delay between colostomy and admission for final repair, was 21 months (range 3 months-11 years, median 12 months, SD 23,5). Thirty-two girls, without a colostomy, had been passing stools through the rectal fistula since birth without any discomfort except one 12 months old female. She had a Recto vestibula thigh fistula, and hard stools distended the abdomen. A woman with a short common channel cloaca was first admitted at the age of 23 years.

F) Type of treatment at birth and complications. Ninety-five (73%) patients out of 130 (M/F ratio 60/35) received a first surgical treatment at a District/Rural Hospital in the neonatal period. In 92 cases, a colostomy was made, including four boys with a low rectoperineal fistula. A blind perineal surgical exploration in seven males and one girl was already described. It was associated with colostomy in five cases. The different types of colonic diversion adopted, and their associated complications and sequelae are reported in Table II. A distal sigmoid colostomy was the most frequently observed (60/92). In about two-thirds of them, there was not enough space between the stomas to exclude the distal loop from faeces. Two stenosed stomas associated with severe constipation were also observed. Inversion of stomas (ten cases, 10,2%) was the first among the visible complications associated with a divided sigmoid colostomy. It was related to an intraoperative twisting of the sigmoid loop. Four of the seven observed prolapses were associated with a Loop colostomy done on sigmoid or transverse colon. Faecal impaction in the distal bowel was always associated with this type of diversion. A loopogram was done in all colostomies and showed a short distal loop in fifteen (16,3%), associated with a misplaced or inverted stoma. Bags were never used in our series, and a rudimental bandage covered stomas. Stoma redoing in twenty-four patients (26%) was mandatory before Posterior sagittal Ano rectoplasty (PSARP) The main indications were Loop colostomy, inverted stoma, short distal loop.

**Table II:** 92 Colostomies – Type, Site, Complications

		Divided	Double Barrel	Loop
Sigmoid	84	60	9	5
*Complications*		*Short distal loop 12* *Inverted stomas 10* *Prolapse 2* *Stenosis of the proximal stoma 2*	*Prolapse 1*	*Short distal* *loop 3* *Prolapse 2*
**Transverse Colon**	6			6
*Complications*				*Prolapse 2*
**L. Colon Flexure**	2		2	
*Complications*			-	

## Discussion

ARM has high relevance in sub Saharan Africa, where Pediatric Surgeons are largely adopting the PSARP approach^9^ that can guarantee a good functional outcome in a high percentage of cases. Outcome depends from a careful clinical and preoperative radiological assessment and a meticulous surgical technique since the first approach. Intraoperative electric stimulation helps to identify the perineal muscular complex. Unfortunately, the number of Pediatric Surgeons is still very few to meet the needs of a Continent where the paediatric population is about half of the total. Few tertiary paediatric Health facilities are concentrated in an urban contest, too far to be accessible by children with a surgical disease living in extra-urban, or rural areas. The majority of them cannot benefit from a specialist treatment since birth, and often they are first referred to the nearest District or Rural Hospital where recommended protocols cannot always be followed. As a consequence, referral is often delayed and a first treatment may frequently result unappropriated or unsatisfactory.

A colostomy is the first emergency procedure to be done in male neonates with ARM within the first 48 hours of life. In females, it can be initially omitted pro-vided that they can evacuate through recto vestibular or vaginal fistula. Some of them can also live in low-in-come countries (LIC) until adulthood without a colostomy and with an unrepaired ARM, even complex, like a 23 years old woman with a Cloaca among our cases.

A divided, sigmoid colostomy is strongly recommended to divert and decompress bowel but also before PSARP to protect the operative site from contamination by faeces^10^. However, a “one-step” approach in neonatal age, without colostomy, has also been proposed and largely debated,, to reduce the surgical load on patients living in LIC as in Africa^11^,^12^.

Although colostomy is generally considered, by non-specialist general surgeons, a simple procedure, some strict rules must be observed in neonates with ARM^8^. The choice of the colonic segmet is crucial. Complications like stoma prolapse or stenosis must be avoided. Loop, double barrel, or even divided colostomies with the two stomas too close each other, do not exclude distal bowel from faeces. Accidental intraoperative twisting of the sigmoid results in stomas inversion and stretching of the distal loop, which makes PSARP of high ARM types somewhat problematic.

Data from our series of patients with ARM referred beyond the neonatal period and, after the first treatment in a non-specialist Hospital, show a high incidence of pitfalls and technical mismanagement. A redoing was required for 26% of colostomies before the final ARM repair. Errors were possibly due to the limited exposition of local practitioners to this anomaly and they mainly regarded the site and the type of stomas. Nevertheless, a transverse loop colostomy, under local Anaesthesia, could have been seen as a faster and lifesaving emergency procedure respect to a divided sigmoid colostomy in a poorly resourced health facility^13^. Blind perineal explorations described in eight cases in our series were also “naïve” attempts to find an alternative to an abdominal procedure. Figures about possible mortality associated with neonatal procedures at District/Rural level are not currently available.

PSARP procedure should follow colostomy as soon as is consented by the patient's condition and after distal loopogram and washes out. In Africa, a delay in the referral to a Pediatric Surgical facility is another crucial issue^7^, ^14^. In Western countries, where most of the deliveries happen in hospitals, an early detection of neonatal surgical emergencies is possible and is followed by an immediate transfer to a Specialist Hub according to shared guidelines. In Africa, like in most of LIC, neonates with a life-threatening but correctable congenital abnormality, can die without being seen by a paediatric surgeon or even recorded. Official figures for their prevalence and mortality rate are so far unreliable and lead to underestimating the real burden of paediatric surgical diseases in LIC respect to Western Counties^15^. A long time may pass between the colostomy doing at birth and the child's referral to a Specialist Pediatric Surgeon^16^. It is the case of three adolescents among our patients, respectively 11,14 and 17 years old.

Delay in referral among our cases could explain why the incidence of associated congenital defects was far below (5.3%) the expected rate (up to 75%) with ARM^17^. In Africa, despite more than 45% of the population is under 14 years of age, there is one Pediatric surgeon for nearly 6,000,000 children^2^. The availability of trained Specialists and the provision of enough Tertiary Healthcare facilities (one for 2,5–3 million population, according to Western Standard)^18^ is still far to be achieved^19^. As an alternative, many elective or urgent paediatric conditions could be managed at a District/Rural Hospital level by practitioners whenever they received an additional training. This model has been suggested for Africa by the WHO^1^ but has not been implemented enough until now. It should require adequate structural investments, effective human resource policy, and a comprehensive Maternal and Child Health Sector Strategy that are still a long-term fix for many under resourced countries. Revisiting Surgical Residents' curriculum to enhance a basic paediatric training could be a first step^20^ to reduce the risk of mismanagement and the burden of deaths or permanent disabilities for many children with treatable congenital disabilities of surgical interest. General surgeons going to practice in a small town or village need only basic paediatric knowledge and practical training (Level-One – General Pediatric Surgery). Surgeons practising in tertiary centres should follow a Level-Two program. A better service to children could be delivered at District/Rural Hospital level by a combination of a local general surgeon with interest in General Pediatric Surgery and a “visiting specialist Pediatric Surgeon” (Outreach). Unfortunately, local specialists' workforce is still too scarce, in sub Saharan Africa, to support these “south-to-south” initiatives.

The adoption of this model is largely justified by the high estimated number of paediatric surgical cases in Africa and has a great convenience for families of children affected by minor surgical conditions seeking for an appropriate care. At the same time the workload of the few specialist centres would be alleviated of those cases not requiring complex treatments. Referral of those who need it could be earlier and safer, following shared guidelines and protocols, promoting closer connections between specialist and peripheral centres.
